# Die Anwendung von Real-world-Evidenz in Entscheidungsprozessen der Arzneimittelregulation

**DOI:** 10.1007/s00103-023-03830-0

**Published:** 2024-01-12

**Authors:** Julia Wicherski, Britta Haenisch

**Affiliations:** 1 Bundesinstitut für Arzneimittel und Medizinprodukte (BfArM), Kurt-Georg-Kiesinger-Allee 3, 53175 Bonn, Deutschland; 2 Deutsches Zentrum für Neurodegenerative Erkrankungen e. V. (DZNE), Bonn, Deutschland; 3 Zentrum für Translationale Medizin, Universität Bonn, Bonn, Deutschland

**Keywords:** Real-world-Evidenz, Real-world-Daten, Arzneimittelregulation, Europäischer Gesundheitsdatenraum, Pharmakoepidemiologie, Real-world evidence, Real-world data, Drug regulation, European health data space, Pharmacoepidemiology

## Abstract

Die Arzneimittelregulation ist ein System zur Förderung und zum Schutz der öffentlichen Gesundheit. Auf dem Markt erhältliche Arzneimittel müssen wirksam, sicher und qualitativ hochwertig sein. Dafür werden von den zuständigen Behörden Entscheidungen auf wissenschaftlicher Basis getroffen. Real-world-Evidenz (RWE) aus Real-world-Daten (RWD) findet bisher überwiegend unterstützende Berücksichtigung bei den Entscheidungsfindungen hinsichtlich der Sicherheit des Arzneimittels nach der Zulassung. Das umfängliche Potenzial von RWE für regulatorische Entscheidungsprozesse entlang des gesamten Produktlebenszyklus wird seit wenigen Jahren zunehmend genutzt und weiter erforscht.

Dieser Beitrag bietet einen Überblick zu aktuellen Anwendungen von RWE in arzneimittelregulatorischen Entscheidungsprozessen. Die diesbezüglichen Potenziale von RWE entlang der zu adressierenden Hürden werden beschrieben sowie Beispiele für aktuelle Projekte zur RWE-Forschung für die Arzneimittelregulation gegeben. Die Arbeit basiert auf aktueller internationaler Literatur sowie Beispielen aus internationalen und europäischen Initiativen und der aktuellen regulatorischen Praxis, die die zunehmende Anwendung von RWD/RWE in regulatorischen Entscheidungsprozessen unterstützen sollen. Um das Potenzial von RWE zukünftig noch besser nutzen zu können, gilt es, durch Forschungsprojekte und Initiativen relevante RWD-Quellen besser verfügbar zu machen, auswertende Methoden weiterzuentwickeln und den Stellenwert von RWE zu etablieren.

## Abstract (en)

Drug regulation is a system to support and protect public health. Drugs with market access must be effective, safe and of high quality. Therefore, drug regulatory decision-making by the competent authorities is made on a scientific basis. Real-world evidence (RWE) from real-world data (RWD) has so far predominantly been taken into account in a supportive manner in drug regulatory decision-making with regard to drug safety after marketing authorisation. The extensive potential of RWE for regulatory decision-making processes along the entire product life cycle has been increasingly used and further examined in recent years.

This article provides an overview of current applications of RWE in drug regulatory decision-making processes. The potentials of RWE along with the hurdles to be addressed are described and examples of current projects on RWE research for drug regulation are given. The work is based on current international literature as well as examples from international and European initiatives and regulatory practice, which aim to support an increased use of RWD/RWE in regulatory decision-making processes. In order to be able to utilise the potential of RWE even more in the future, it is important to make relevant RWD sources more readily available through research projects and initiatives, to further develop evaluative methods and to establish the significance of RWE.

## Hintergrund

Um die Wirksamkeit, Sicherheit und Qualität von Arzneimitteln zu gewährleisten und damit die öffentliche Gesundheit zu schützen, werden im Rahmen der Arzneimittelregulation von den zuständigen Behörden Entscheidungen auf wissenschaftlicher Basis getroffen. Über den gesamten Produktlebenszyklus werden evidenzbasierte Informationen aus verschiedenen Datenquellen in die Prozesse der Entscheidungsfindung einbezogen. Dabei werden vor allem Daten aus randomisierten klinischen Studien („randomised controlled trials“ [RCTs]) und Real-world-Daten (RWD) zur Generierung von Real-world-Evidenz (RWE) verwendet. In der evidenzbasierten Medizin gelten RCTs als Goldstandard, um neues Wissen zu generieren. Dies gilt auch, wenn für Arzneimittel unter kontrollierten Bedingungen die Wirksamkeit und Sicherheit ermittelt werden soll [1, 2].

RWE und RWD sind relativ moderne Begriffe, die einschlägig von den Arzneimittelbehörden und weiteren Stakeholdern genutzt und definiert werden. Drei sehr bekannte Definitionen stammen von der europäischen Arzneimittelbehörde „European Medicines Agency“ (EMA), der US-amerikanischen Arzneimittelbehörde „U.S. Food and Drug Administration“ (FDA) und der gemeinnützigen globalen „Professional Society for Health Economics and Outcomes Research“ (ISPOR). Laut allen drei Institutionen bilden RWD vielfältige Informationsquellen zum Gesundheitszustand und zur Gesundheitsversorgung der Bevölkerung [3–5]. Die EMA definiert RWD wörtlich als jegliche „routinemäßig erfassten Daten zum Gesundheitszustand oder der Nutzung von Gesundheitsleistungen, die nicht aus RCTs stammen“ [3]. Auch die ISPOR-Definition grenzt RWD als Daten, die nicht aus konventionellen RCTs stammen, ab. Die FDA bleibt in diesem Punkt offener und schließt RCTs nicht wortwörtlich aus der Definition von RWD aus.

RWD umfassen sämtliche gesundheitsbezogenen Daten. Dabei gibt es eine große Spannweite von Studiendesigns und Datenquellen. Diese erstreckt sich von Querschnittserhebungen und Gesundheitsapplikationen über Versicherungs- und Registerdaten sowie die elektronische Patient:innenakte bis hin zu epidemiologischen/klinischen primärdatenbasierten Kohortenstudien und nichtrandomisierten klinischen Studien, wie pragmatischen oder einarmigen klinischen Studien. Auch hybride Studiendesigns oder Studien mit mehreren stufenartig angeordneten Designs sind möglich [6, 7]. RWD sind damit ergänzend zu den RCTs eine sehr wichtige Wissensquelle zur Einschätzung der erwünschten und unerwünschten Wirkungen, Folgen und Rahmenbedingungen eines Arzneimittels unter alltäglichen/realen Anwendungsbedingungen. Der aus RWE erfasste Informationsgehalt wird in der Arzneimittelregulation bislang schwerpunktmäßig in dem Produktlebenszyklusschritt nach der Zulassung („post-authorisation phase“ oder „post-marketing surveillance“), der Überwachung von unerwünschten Arzneimittelwirkungen (UAW) und der Re-Evaluation/Erweiterung des Indikationsspektrums etabliert.

Beide Evidenzquellen, RWD und RCTs, sind aufgrund ihrer natürlichen Erhebungsmethoden durch spezifische Stärken und Limitationen charakterisiert. Diese sind insbesondere abhängig von dem jeweiligen Untersuchungsziel einer Studie. Für jede Untersuchung ist es daher wichtig, das Ziel genau zu definieren sowie das am besten geeignete Studiendesign und die passende Studienpopulation auszuwählen. Limitationen einzelner Evidenzquellen lassen sich zudem – bei komplementärer/gemeinschaftlicher Verwendung von RWD und RCT – größtenteils gegenseitig adressieren und die Aussagekraft entsprechend erhöhen. Darüber hinaus bieten RWD unter der Anwendung entsprechend geeigneter Methoden zur Gewinnung der RWE das große Potenzial, einen Beitrag zum Schließen aktueller Informationslücken im Produktlebenszyklusschritt vor und während der Zulassung/Evaluation eines Arzneimittels zu leisten, insbesondere in Situationen, in denen RCTs aus ökonomischen, pragmatischen und/oder ethischen Gründen nicht zu rechtfertigen sind [8–10].

Zwei exemplarische Arzneimittel, bei denen RWD die Zulassungen bei fehlenden RCTs deutlich unterstützten, sind Blinatumomab und Comirnaty® (Biontech, Mainz, Deutschland, Pfizer, New York City, NY, USA)/Vaxzevria® (AstraZeneca, Cambridge, UK) [11]. Blinatumomab (Blincyto®, Amgen, Thousand Oaks, CA, USA) ist ein Medikament zur Behandlung der akuten lymphatischen Leukämie, einer seltenen und schwerwiegenden Erkrankung mit schlechter Prognose und wenigen Behandlungsoptionen. Als RWD wurde eine historische Vergleichsgruppe bestehend aus gepoolten Daten klinischer Zentren aus Europa, dem Vereinigten Königreich und den USA verwendet. Die Analyse dieser historischen Vergleichsgruppe komplementierte die einarmige klinische Studie und trug somit zur Beschleunigung des Zulassungsentscheidungsprozesses bei. Untersucht wurden die vollständige Remission und die gesamte Überlebenszeit unter der Anwendung des Behandlungsstandards der Salvage-Chemotherapie im Vergleich zur neuartigen Therapie mit Blinatumomab [11, 12].

Die SARS-CoV-2-Pandemie ist das zweite, sehr aktuelle Beispiel für Situationen, in denen sehr schnelle Entscheidungen (ggf. auch für spezifische Subpopulationen) benötigt werden. Im Zulassungsfall der beiden Impfstoffe Comirnaty® und Vaxzevria® waren die vorhandenen RCTs zu unspezifisch oder lückenhaft hinsichtlich ihrer Aussagekraft zur Wirksamkeit bei über 60-jährigen Personen und es fehlte die Zeit, ein neues RCT durchzuführen. Stattdessen wurden elektronische Gesundheitsaufzeichnungen („electronic health records“ [EHR]) aus Schottland, bestehend aus Daten der Primärversorgung, Labordaten, Daten aus Krankenhausaufenthalten und dem Sterberegister, mit den Impfdaten verknüpft, um zeitnah eine prospektive Kohortenstudie in Echtzeit durchführen zu können [11, 13]. Die EMA [14] unterstützt Forschung zur methodischen Weiterentwicklung der Analyse von RWD zum komplementären Einsatz von RWE in allen Schritten der Arzneimittelregulation.

## Aktuelle Anwendung von RWE in Entscheidungsprozessen

Die Anwendung von RWE in der Arzneimittelregulation in Europa und den USA der letzten Jahre wurde bereits von mehreren Wissenschaftler:innen untersucht [15–17] und zusammengefasst [18]. Das einheitliche Fazit ist, dass RWD bereits in allen Schritten der Arzneimittelregulation Berücksichtigung findet, insbesondere jedoch in der etablierten Post-Marketing-Surveillance [18]. Bei der EMA wurden von Januar 2018 bis Dezember 2019 insgesamt 111 Neuzulassungen beschlossen. Bei allen Zulassungen wurde irgendeine Form von RWE einbezogen. In über 80 % der Zulassungen wurde RWE für die Beschreibung der Sicherheitsprofile des Arzneimittels verwendet. Nur bei 33 % der Zulassungen wurde die Evidenz aus RWD durch eigene Analysen selbst generiert [15]. In den USA gab es 136 Neuzulassungen im Zeitraum Januar 2019 bis Juni 2021, von denen 116 RWE enthielten. Der Nachweis von Wirksamkeit und Sicherheit war in 76 % der Zulassungsunterlagen Grund für den Einsatz von RWE. In 56 % der Neuzulassungen wurde die enthaltene RWE bei der Entscheidungsfindung berücksichtigt [17].

Jüngst publizierte die EMA einen neuen Report zu den 61 erfassten Pilotstudien zur RWE-Generierung im Zeitraum 01.09.2021 bis 07.02.2023 [14]. 36 Studien wurden als umsetzbar/durchführbar hinsichtlich Verfügbarkeit der relevanten Daten und des zeitlichen Horizonts eingeschätzt (darunter 27 Studien bereits abgeschlossen), 19 Studien wurden als nicht umsetzbar eingeschätzt und 6 Studien befanden sich noch in Beurteilung oder pausierten. Der Großteil der Studien wurde über der EMA zur Verfügung stehende Datenbanken umgesetzt, lediglich 8 Studien über das Netzwerk DARWIN EU®^1^ und weitere 4 Studien über das EMA-Rahmenkonzept (FWC). Für den Ausschuss für Risikobewertung im Bereich der Pharmakovigilanz (PRAC) und den Pädiatrieausschuss (PDCO) wurden mit 28 bzw. 14 Studien die meisten Studien durchgeführt. Insgesamt 19 der abgeschlossenen Studien wurden binnen 90 Tagen abgeschlossen, der überwiegende Anteil dieser Studien wurde mit Daten aus Deutschland, Frankreich und dem Vereinigten Königreich durchgeführt. In einer zusätzlichen Befragung innerhalb des Netzwerks empfand die Mehrheit die abgeschlossenen Studien als hilfreich/unterstützend für die eigenen Einschätzungen [14].

## Das Potenzial von RWE-Forschung für die Arzneimittelregulation

Zusammengefasst liegt das größte Potenzial von RWE für die Arzneimittelregulation in der zeit-, kosten- und patient:innenschonenden Erfassung und Verarbeitung gesundheitsbezogener Informationen für eine schnelle und bevölkerungsrepräsentative Berücksichtigung in regulatorischen Entscheidungsprozessen. Insbesondere im Hinblick auf die aktuellen Gegebenheiten und Herausforderungen in der Gesundheitsversorgung, beispielsweise durch zunehmende Globalisierung, den Klimawandel und die SARS-CoV-2-Pandemie, ist es von hoher gesundheitswissenschaftlicher und gesundheitspolitischer Relevanz, qualitativ hochwertige, große, standardisierte Forschungsdatenbanken zu etablieren, um valide Vorhersagemodelle und akute Situationseinschätzungen im Sinne des Gesundheitsschutzes der Bevölkerung umsetzen zu können, wenn sie benötigt werden. Konkrete Anwendungsbereiche, in denen das Potenzial von RWD bereits zunehmend Anwendung findet, weil RCTs nicht vertretbar bzw. nicht möglich sind [3], sind unter anderem die Erforschung seltener Erkrankungen, Erkrankungen mit einhergehender sehr hoher Vulnerabilität der Patient:innen, unvorhersehbare akute Situationen oder Erkrankungsbilder ohne Behandlungsalternativen.

Perspektivisch liegt ein weiteres Potenzial der RWE-Forschung für die Arzneimittelregulation in den diversen RWD-Quellen selbst. Sie bieten eine große Auswahl passender Informationsquellen für spezifische Fragestellungen. Um dieses Potenzial nutzen zu können, sollten zunächst die Hürden zu Akzeptanz und Verständnis dieser Datendiversität überwunden werden. Es ist wichtig, Wissen bei Forschenden und Regulator:innen darüber zu vermitteln, was RWD per Definition sind und dass je nach RWD-Quelle andere Methoden und eine andere Aussagekraft oder ein anderer Einsatzbereich resultieren, damit das datenquellenspezifische Potenzial eines realistischen komplementären Beitrags von RWE neben RCTs für die Entscheidungsprozesse genutzt werden kann. Auch in der Versorgungspraxis besteht unter den gesundheitsdienstleistungserbringenden, -erhaltenden und -finanzierenden Personen der Bedarf an zielgruppenorientierter Wissensvermittlung, um Akzeptanz und Verständnis bzgl. RWD zu verbessern. Das Wissen um die Bedeutung der sich selbst generierenden Datenschätze und deren Relevanz für die Patient:innensicherheit und damit die Bereitschaft zur Datenspende sowie Motivation und Zeitaufwand zur Dokumentation der gesundheitsbezogenen Daten im Praxisalltag sind derzeit noch eingeschränkt und müssen adressiert werden.

Die einfache Anwendbarkeit und Erreichbarkeit von zentral organisierten, standardisierten Datenbanken ist eine weitere Möglichkeit, arzneimittelregulatorische Entscheidungsprozesse zu unterstützen. Dabei ist es besonders wichtig, relevante und aussagekräftige Datenquellen schnell aufzufinden und auch möglichst rasch und flexibel nutzen zu können. Zudem werden viele fortgeschrittene Methoden wie der Einsatz von künstlicher Intelligenz und maschinellem Lernen in der Datenaufbereitung und -analyse als großes Potenzial für die zukünftige RWE-Generierung betrachtet.

## RWE-Initiativen und aktuelle RWE-Forschungsprojekte für die Arzneimittelregulation

Im Folgenden werden einige wichtige RWE-Initiativen und -Forschungsprojekte für die Arzneimittelregulation exemplarisch dargestellt.

Der europäische Gesundheitsdatenraum (European Health Data Space [EHDS]) ist ein wichtiger Bestandteil der europäischen Gesundheitsdateninfrastruktur. Hier wird an der Verbesserung der digitalen Nutzbarkeit von Gesundheitsdaten in Europa gearbeitet mit dem Ziel einer EU-weiten Verfügbarkeit von Gesundheitsdaten für die „Schaffung eines kohärenten, vertrauenswürdigen und effizienten Rahmens für die Nutzung von Gesundheitsdaten für Forschung, Innovation, Politikgestaltung und Regulierung“ [18, 19]. Von regulatorischer Seite ist die Vision, dass bis zum Jahr 2025 die Nutzung von RWE ermöglicht und der Wert für das gesamte Spektrum des Produktlebenszyklus (vor und nach der Zulassung) der regulatorischen Anwendungsfälle nachgewiesen sein wird [18, 20].

DARWIN EU® leistet einen wichtigen Beitrag zur RWE-gestützten Entscheidungsfindung in der Arzneimittelregulation. DARWIN EU® pflegt einen Datenquellenkatalog, stellt hochqualitative validierte RWD-Quellen zur Verfügung und beschäftigt sich mit relevanten Fragestellungen rund um das Thema RWD-Studien [21]. Dabei ist es vorgesehen, dass durch die Etablierung von DARWIN EU® bis 2025 mehr als 100 Studien pro Jahr über dieses Netzwerk verwirklicht werden sollen [22]. Zur Umsetzung der DARWIN EU®-Ziele und zur Unterstützung der Europäischen Kommission beim Aufbau des EHDS gab es initial die Initiative „Joint Action Towards the European Health Data Space“ (TEHDAS) [23].

Zur Pilotierung der Infrastruktur des EHDS für die Sekundärnutzung von Gesundheitsdaten wurde das Projekt „Health Data @ EU Pilot“^2^ gestartet. Das Projekt soll Datenplattformen in einer Netzinfrastruktur verbinden und somit Forschungsprojekte unterstützen, die Gesundheitsdaten aus verschiedenen EU-Mitgliedstaaten nutzen. Ebenfalls werden Leitlinien für Datenstandards, Datenqualität, Datensicherheit und Datentransfer zur Unterstützung dieser grenzüberschreitenden Infrastruktur bereitgestellt. Dazu sollen ein Metadaten-Suchdienst und eine gemeinsame Anfrage für den Zugang zu Gesundheitsdaten entwickelt werden. Zur Veranschaulichung der Machbarkeit und des Potenzials der Sekundärdatennutzung in mehreren europäischen Ländern wird das Projekt fünf regulatorisch relevante Anwendungsfälle einbeziehen.

Die EMA positioniert sich zudem in einem Kommentar im British Medical Journal (BMJ) dazu, dass RWD weiterhin als Ergänzung komplementär zu RCTs verwendet werden sollten und ausdrücklich nicht als Alternative zu RCTs [24]. Die Autoren Daniel Morales und Peter Arlett schreiben, dass RCTs aus Sicht der EMA weiterhin das beste Studiendesign zur Beantwortung vieler Fragestellungen (insbesondere für den primären Nachweis der Wirksamkeit neuer Medikamente) sind und bleiben. RWE seien dennoch essentiell für andere Fragestellungen, wie etwa zur Epidemiologie von Erkrankungen, zur Arzneimittelanwendung und zu Sicherheitsstudien. Neben den Bemühungen, die Verwendung von RWD weiter auszubauen, wie beispielsweise durch die beschriebene Initiative DARWIN EU®, sollen RCTs verbessert werden. Dies unterstützt die EMA zum Beispiel durch die Implementierung der „Clinical Trial Regulation“ und die Initiative „Accelerating Clinical Trials in the EU“ (ACT EU). Abschließend regen Morales und Arlett an, Definitionen und Begrifflichkeiten rund um das Thema RWD/RWE zu harmonisieren. Die EMA wird dazu die Erstellung eines internationalen Leitfadens unterstützen [24].

Ein entscheidender Faktor, der dazu beiträgt, dass RWE aus RWD dauerhaft als zuverlässige Quelle in der regulatorischen Entscheidungsfindung berücksichtigt wird, ist die Weiterentwicklung der Methodik zur Auswertung von RWD für verschiedene regulatorische Anwendungsfälle. Das dafür notwendige Wissen sollte anhand realer Anwendungsfälle (und deren methodischer Grenzen) generiert werden. Deshalb starteten im Jahr 2023 fünf europäische Projekte im Rahmen der Ausschreibung durch Horizon Europe unter dem Titel „New methods for the effective use of real-world data and/or synthetic data in regulatory decision-making and/or in health technology assessment“ (ID HORIZON-HLTH-2022-TOOL-11-02). Diese Projekte heißen „More-EUROPA“, „ONCOVALUE“, „REALM“, „REDDIE“ und „Real4Reg“. Das übergeordnete Ziel dieser Projekte, die sich als Gruppe der geförderten Projekte unter dem Namen „MetReal Cluster“ regelmäßig austauschen, ist es, datengesteuerte Methoden zur effektiven Nutzung von RWD (inkl. synthetischer Daten und maschinellen Lernens) für die regulatorischen Entscheidungsprozesse zu entwickeln und an anwendungsbezogenen Beispielen zu etablieren. Das MetReal Cluster soll gemeinsame Aktivitäten und den Austausch von Erfahrungen zwischen den Projekten fördern und daraus entstehende Synergien unterstützen. More-EUROPA^3^ setzt dabei den individuellen Schwerpunkt auf die Nutzung von Registerdaten zur Berücksichtigung von Patient:innen-zentrierten/-berichteten Outcomes und deren Messung im Bereich Krebs, Multiple Sklerose und Herzinsuffizienz. ONCOVALUE^4^ fokussiert sich auf die Echtzeitanalyse von RWD in onkologischen Kliniken, um die Versorgung von Krebspatient:innen und die Einschätzung neuer Therapieansätze weiter verbessern zu können. REALM^5^ konzentriert sich auf die Entwicklung innovativer Software für Medizinprodukte. REDDIE^6^ setzt den Schwerpunkt auf die Diabetesforschung und die Emulation von Zielparametern von RCTs. Real4Reg^7^ wird vom BfArM koordiniert, es möchte die Nutzbarkeit von RWD im gesamten Produktlebenszyklus anhand verschiedener Anwendungsbeispiele verbessern.

Im Real4Reg-Konsortium arbeiten zehn europäische Partner aus regulatorischen Institutionen, akademischen Einrichtungen und Patientenorganisationen aus sechs EU-Ländern zusammen. Dabei werden heterogene Gesundheitsdaten aus Dänemark, Finnland, Portugal und Deutschland ausgewertet. Auch das Forschungsdatenzentrum Gesundheit (FDZ Gesundheit) beteiligt sich als ein Bereitsteller von RWD am Projekt. Die für das Projekt getroffene Auswahl an Anwendungsfällen beinhaltet für die Schritte im Produktlebenszyklus vor der Zulassung („pre-authorisation“) die Potenziale und Herausforderungen beim Einsatz von RWE bei den Erkrankungen Amyotrophe Lateralsklerose (ALS) und Brustkrebs sowie für die Schritte nach der Zulassung („post-authorisation“) die Anwendung von RWE zur Beurteilung der Sicherheit von Fluorchinolonen und zu möglichen Erweiterungen des Indikationsspektrums von Natrium-Glukose-Kotransporter-2(SGLT-2)-Inhibitoren. Unterstützt wird das Konsortium durch die Expertise des Advisory Bords, welches Repräsentant:innen von Regulierungsbehörden, Krankenkassen, Patient:innenorganisationen, aus der klinischen Versorgung und der pharmazeutischen Industrie einschließt. Die im Real4Reg-Projekt entwickelten Lösungen für die RWD-Analyse zur regulatorischen Entscheidungsfindung werden dabei helfen, neue und optimierte Leitliniendokumente und Schulungskonzepte zu definieren, die potenziell über den gesamten Produktlebenszyklus und in allen EU-Ländern angewendet werden können.

Der Einsatz von künstlicher Intelligenz in den Analysen und die Entwicklung von Analyseanwendungen können dazu beitragen, dass zukünftig mehr standardisierte RWD-Studien durchgeführt werden. Der Einsatz dieser Analyseanwendungen wiederum beschleunigt die Analyse der Daten und erleichtert die Interpretation und Bewertung dieser Studien für die Regulator:innen. Weiterhin unterstützt wird die Etablierung von RWE über ein geplantes Trainingskonzept, welches Regulator:innen dazu befähigen und es erleichtern soll, die Einbeziehung von RWE in regulatorische Entscheidungen zu bewerten. Dies trägt auch zur verstärkten Nutzung von RWE im EHDS bei.

Die wachsende Bedeutung von RWE in der Arzneimittelregulation ist auch aus der Erstellung von Handlungsempfehlungen durch übergeordnete Institutionen des Gesundheitswesens ersichtlich. So hat die Arbeitsgruppe XIII „Real-World Data and Real-World Evidence in Regulatory Decision Making“ des „Rates für internationale Organisationen der medizinischen Wissenschaft“ (Council for International Organizations of Medical Sciences [CIOMS]) gerade ein umfassendes Dokument zur Anwendung von RWD und RWE in der regulatorischen Entscheidungsfindung erstellt, welches bereits die öffentliche Konsultation durchlaufen hat [18, 25].

## Fazit

RWE steht vor einer Veränderung, was ihre Verwendung und Akzeptanz bei der regulatorischen Entscheidungsfindung angeht. Sowohl aufgrund der erleichterten Verfügbarkeit von RWD national sowie über Initiativen, wie Darwin EU, als auch bedingt durch aktuelle Situationen, wie die SARS-CoV-2-Pandemie, hat die Relevanz von RWE für die Arzneimittelregulation deutlich zugenommen. Zudem erhöhen die Möglichkeiten zur Datenqualitätssicherung die Akzeptanz dieser Daten. Neben der klassischen Anwendung im Post-authorisation-Bereich, vornehmlich in der Pharmakovigilanz, ermutigen Anwendungsbeispiele zunehmend den Einsatz von RWE in allen Phasen des Produktlebenszyklus. Dabei gibt es zahlreiche Herausforderungen zu meistern, vor allem hinsichtlich einer Verbesserung von Methodik, Datenqualität, Transparenz und Verständnis von möglichen Restverzerrungen. Daneben sollte trotz aller Potenziale von RWE berücksichtigt werden, dass RWD aktuell klinische Studien weder ersetzen noch überflüssig machen. Um die Evidenz von RWD zu erhöhen, sollten Maßnahmen zur Verbesserung der Interpretierbarkeit und Verlässlichkeit von RWD einbezogen werden, z. B. die Standardisierung von Datenqualitäten und die Erstellung von Studienprotokollen und statistischen Analyseplänen. Um zu einer angemessenen Sichtweise gegenüber Methoden zur Generierung von RWE zu gelangen, sind kontinuierliche Forschung und der stetige Austausch über Methoden und Ergebnisse erforderlich.

## Footnotes

1 DARWIN EU® (Data Analysis and Real World Interrogation Network) ist das Koordinierungszentrum der EMA und des europäischen medizinregulatorischen Netzwerks (EMRN) zur zeitnahen Bereitstellung zuverlässiger Evidenz aus Gesundheitsdatenbanken der EU für die Verwendung, Sicherheit und Wirksamkeit von Arzneimitteln.

2 EHDS-Health Data@EU Pilot. https://ehds2pilot.eu/; Zugegriffen: 28. August 2023.

3 More-EUROPA: https://umcgresearch.org/more-europa/; Zugegriffen: 22. November 2023.

4 ONCOVALUE—Implementing value-based oncology care at European cancer hospitals: https://oncovalue.org/; Zugegriffen: 22. November 2023.

5 REALM—Innovative solutions for the creation and evaluation of medical device software: https://www.realm-ai.eu/; Zugegriffen: 22. November 2023.

6 REDDIE—Real-World Evidence for Decisions in Diabetes: https://www.reddie-diabetes.eu/; Zugegriffen: 22. November 2023.

7 Real4Reg—Use cases for development, optimisation and implementation of artificial intelligence methods for real world data analyses in regulatory decision-making and health technology assessment along the product lifecycle: https://www.real4reg.eu/; Zugegriffen: 28. August 2023.

## References

[1] Britton A, Mckee M, Black N (1998). Choosing between randomised and non-randomised studies: a systematic review. Health Technol Assess.

[2] Jüni P, Altman DG, Egger M (2001). Assessing the quality of controlled clinical trials. BMJ.

[3] Cave A, Kurz X, Arlett P (2019). Real-world data for regulatory decision making: challenges and possible solutions for Europe. Clin Pharmacol Ther.

[4] U.S. Food and Drug Administration (FDA) (2018) Framework for FDA‘s Real-World Evidence Program. https://www.fda.gov/media/120060/download?attachment. Zugegriffen: 2. Jan. 2024

[5] Garrison LP Jr, Neumann PJ, Erickson P, Marshall D, Mullins CD (2007) Using real-world data for coverage and payment decisions: the ISPOR Real-World Data Task Force report. Value Health 10(5):326–335 10.1111/j.1524-4733.2007.00186.x 17888097

[6] European Medicines Agency (EMA) (2022) Good practice guide for the use of the metadata catalogue of real-world data sources, V1.0. https://www.ema.europa.eu/en/documents/regulatory-procedural-guideline/good-practice-guide-use-metadata-catalogue-real-world-data-sources_en.pdf. Zugegriffen: 28. Aug. 2023

[7] Getreal (2020) RWE navigator: Sources of RWD. https://rwe-navigator.eu/use-real-world-evidence/sources-of-real-world-data/. Zugegriffen: 28. Aug. 2023

[8] European Medicines Agency (EMA) (2016) Scientific guidance on post-authorisation efficacy studies. https://www.ema.europa.eu/en/documents/scientific-guideline/scientific-guidance-post-authorisation-efficacy-studies-first-version_en.pdf. Zugegriffen: 28. Aug. 2023

[9] Baumfeld Andre E, Reynolds R, Caubel P (2020). Trial designs using real-world data: the changing landscape of the regulatory approval process. Pharmacoepidemiol Drug Saf.

[10] Eichler H-G, Pignatti F, Schwarzer-Daum B (2021). Randomized controlled trials versus real world evidence: Neither magic nor myth. Clin Pharmacol Ther.

[11] Spooner Á (2023) European Medicines Agency (EMA) Multi-stakeholder workshop on Real World Data (RWD) quality and Real-World Evidence (RWE) use. Use of RWE in medicines development and regulatory submissions—an industry perspective. https://www.ema.europa.eu/en/documents/presentation/presentation-use-rwe-medicines-development-regulatory-submissions-industry-perspective-spooner_en.pdf. Zugegriffen: 22. Nov. 2023

[12] Gökbuget N, Kelsh M, Chia V, Advani A, Bassan R, Dombret H, Doubek M, Fielding AK, Giebel S, Haddad V, Hoelzer D, Holland C, Ifrah N, Katz A, Maniar T, Martinelli G, Morgades M, O’Brien S, Ribera JM, Rowe JM, Stein A, Topp M, Wadleigh M, Kantarjian H (2016). Blinatumomab vs historical standard therapy of adult relapsed/refractory acute lymphoblastic leukemia. Blood Cancer J.

[13] Vasileiou E, Simpson CR, Shi T, Kerr S, Agrawal U, Akbari A, Bedston S, Beggs J, Bradley D, Chuter A, de Lusignan S, Docherty AB, Ford D, Hobbs FDR, Joy M, Katikireddi SV, Marple J, McCowan C, McGagh D, McMenamin J, Moore E, Murray JLK, Pan J, Ritchie L, Shah SA, Stock S, Torabi F, Tsang RSM, Wood R, Woolhouse M, Robertson C, Sheikh A (2021). Interim findings from first-dose mass COVID-19 vaccination roll-out and COVID-19 hospital admissions in Scotland: a national prospective cohort study. Lancet.

[14] European Medicines Agency (2023) Real-world evidence framework to support EU regulatory decision-making: Report on the experience gained with regulator-led studies from September 2021 to February 2023, EMA/289699/2023. https://www.ema.europa.eu/en/documents/report/real-world-evidence-framework-support-eu-regulatory-decision-making-report-experience-gained_en.pdf. Zugegriffen: 28. Aug. 2023

[15] Eskola SM, Leufkens HGM, Bate A (2022). Use of real world data and evidence in drug development of medicinal products centrally authorized in europe in 2018–2019. Clin Pharmacol Ther.

[16] Flynn R, Plueschke K, Quinten C (2022). Marketing authorization applications made to the European medicines agency in 2018–2019: What was the contribution of real-world evidence?. Clin Pharmacol Ther.

[17] Purpura CA, Garry EM, Honig N (2022). The role of real-world evidence in FDA-approved new drug and biologics license applications. Clin Pharmacol Ther.

[18] Wicherski J, Schneider K, Zinserling J, Heß S, Haenisch B, Broich K (2023). Real-world-Daten in der Arzneimittelregulation – aktuelle Entwicklungen und Ausblick. Präv Gesundheitsf.

[19] Europäische Kommission https://ec.europa.eu/health/ehealth-digital-health-and-care/european-health-data-space_de. Zugegriffen: 28. Aug. 2023

[20] Arlett P, Kjær J, Broich K (2022). Real-world evidence in EU medicines regulation: enabling use and establishing value. Clin Pharmacol Ther.

[21] European Medicines Agency (2022) Data Analysis and Real World Interrogation Network (DARWIN EU). https://www.ema.europa.eu/en/about-us/how-we-work/big-data/data-analysis-real-world-interrogation-network-darwin-eu. Zugegriffen: 28. Aug. 2023

[22] Big data use for public health. https://www.ema.europa.eu/en/news/big-data-use-public-health-publication-big-data-steering-group-workplan-2022-25. Zugegriffen: 28. Aug. 2023

[23] Finnish innovation fund Sitra joint action towards the European health data space—TEHDAS. https://tehdas.eu/. Zugegriffen: 28. Aug. 2023

[24] Morales D, Arlett P (2023). RCTs and real world evidence are complementary, not alternatives. BMJ.

[25] Council for International Organizations of Medical Sciences (CIOMS) Working group XIII Real-world data and real-world evidence in regulatory decision making. https://cioms.ch/working-groups/real-world-data-and-real-world-evidence-in-regulatory-decision-making/. Zugegriffen: 28. Aug. 2023 10.1002/pds.70117 PMC11897686 40070104

